# Prototype of an Innovative Vacuum Dryer with an Ejector System: Comparative Drying Analysis with a Vacuum Dryer with a Vacuum Pump on Selected Fruits

**DOI:** 10.3390/foods12173198

**Published:** 2023-08-25

**Authors:** Zdravko Šumić, Aleksandra Tepić Horecki, Vladimir Kašiković, Andreja Rajković, Lato Pezo, Tatjana Daničić, Branimir Pavlić, Anita Milić

**Affiliations:** 1 Faculty of Technology Novi Sad, University of Novi Sad, Bulevar cara Lazara 1, 21000 Novi Sad, Serbia; sumic@uns.ac.rs (Z.Š.);; 2 Independent Researcher, 21000 Novi Sad, Serbia; 3 Research Unit Food Microbiology and Food Preservation, Department of Food Technology, Safety and Health, Faculty Bio-Science Engineering, Ghent University, 9000 Ghent, Belgium; 4 Institute of General and Physical Chemistry, University of Belgrade, 11000 Belgrade, Serbia

**Keywords:** vacuum dryer, prototype, ejector system, physico-chemical properties, energy efficiency

## Abstract

The following article describes new research about the design, construction and installation of the new prototype of a vacuum dryer with an ejector system. Moreover, the testing of this new prototype involved comparing the qualities of fruit dried in a vacuum drier with an ejector system to fruit dried in a convectional vacuum drier. The data obtained were then analyzed and presented. Due to their economic relevance and highly valuable nutritional value and sensory properties, sour cherries and apricots have been chosen to be the subjects for the testing. The most appropriate quality indicators for analyzing were moisture content, a_w_ value, share and penetration force, total phenol, flavonoid and anthocyanin content and antioxidant activity (FRAP, DPPH and ABTS test). The main results of this study were achieved by designing, constructing, installing and testing the usage of the innovative prototype of a vacuum dryer with an ejector system in the laboratory of the Technology of fruit and vegetable products of the Faculty of Technology Novi Sad, University of Novi Sad. Based on our analyses of the obtained data, it was concluded that vacuum dryer with an ejector system are similar to vacuum dryer with a vacuum pump in terms of all tested physical, chemical and biological properties of dried samples. We observed similarities in some of the most important parameters, including product safety and quality, such as the a_w_ value and the total phenol content, respectively. For example, in dried sour cherry, the a_w_ values ranged from 0.250 to 0.521 with the vacuum pump and from 0.232 to 0.417 with the ejector system; the total phenol content ranged from 2322 to 2765 mg GAE/100 g DW with the vacuum pump and from 2327 to 2617 mg GAE/100 g DW with the ejector system. In dried apricot, the a_w_ ranged from 0.176 to 0.405 with the vacuum pump and from 0.166 to 0.313 with the ejector system; total phenol content ranged from 392 to 439 mg GAE/100 g DW with the vacuum pump and from 378 to 428 mg GAE/100 g DW with the ejector system.

## 1. Introduction

The most common techniques for drying fruit include solar drying, convective drying and conduction drying. The disadvantages of sun drying and convective drying in terms of the presence of oxygen during the drying process could be successfully overcome using vacuum drying, one of the techniques of conduction drying. Vacuum drying takes place in an atmosphere with a very low oxygen content, and thus it could be performed at lower temperatures than those used in convective drying. During the drying process under these conditions, undesirable oxidation in dried material is significantly slowed down, which further positively affects the preservation of important bioactive substances naturally present in fresh fruit. Different innovative fruit and vegetable drying technologies have been investigated in detail, such as multi-flash drying, microwave vacuum drying of pumpkins [1] and ultrasound-assisted vacuum drying of nectarines [2]. Microwave vacuum drying was also investigated in detail by Raj and Dash [3], where dragon fruit slices were dried through microwave vacuum drying. The effect of ultrasound pretreatment and microwave vacuum drying in the production of dried poniol fruit was investigated by Dash et al. [4], while low-temperature vacuum drying as a novel process to improve papaya (*Vasconcellea pubescens*) nutritional and functional properties was presented in a paper by Vega-Gálvez et al. [5].

The most established and most often used fruit drying technology is convective drying, which has many technical and technological disadvantages [6], such as high drying temperatures, uneven quality of dried products, significant manual labor requirements for maintenance etc. The results of drying in these dryers are dried products that, due to their rather poor physical, chemical and biological properties, do not meet market quality requirements. However, there are a small number of dryers, such asclassic vacuum dryers or lyophilizators that work on the principle of vacuum drying and produce high quality dried fruit [7]. The main disadvantages of these dryers are their limited capacity, the very high investment in the equipment and the high maintenance costs of the equipment. There is a constant need for improvements in the field of fruit drying in order to overcome these shortcomings.

The ejector system has been widely and successfully applied in many different areas and in different fields, such as, for example, the application of a gas-liquid ejector to a solar-powered bi-ejector refrigeration system described in a paper by Shen et al. [8]. Recent work conducted by Śmierciew et al. [9] elaborated on the engineering application of numerical modelling for the design of the gas ejector operating in the ejection refrigeration system driven by a low-temperature heat source. Regarding the application of ejector systems in the field of food drying, Suryanto et al. [10] described novel vacuum drying using the steam ejector applied to cocoa and coffee beans.

Serbia is a country with a favorable temperate climate, which provides suitable growing conditions for different types of continental stone fruits [11]. Sour cherry (*Prunus cerasus*) and apricot (*Prunus armeniaca*) are among the most common stone fruits in Serbia with significant commercial importance [12]. Apart from their different chemical compositions, properties and use, both crop types are members of the genus *Prunus*, from the stone fruit family *Rosaceae* [13]. In addition to other stone fruit types, sour cherries and apricots have been the subject of comprehensive research by many authors due to their rich chemical composition and desirable sensory properties [14,15,16,17,18,19]. A close link between the present bioactive compounds and their positive impact on human health has been established [20,21].

To summarize all of the above, on the one hand, searching for a solution to the problem of high vacuum pump costs and investments and, on the other hand, obtaining a high-quality dried product, the main idea was to replace the vacuum pump with an ejector system in order to meet the previously mentioned conditions. Since there are few papers published that describe in detail the application of an ejector system to a vacuum dryer for fruit and vegetable drying, the main goal of this research was to make a significant contribution in the field of fruit drying in terms of reducing investment costs in vacuum drying equipment and maintenance costs, as well as in terms of the possibility of producing dried fruit of very good quality, equal to or better than dried fruit produced using a vacuum dryer with a vacuum pump. The limit regarding the sort of fruit that could be dried in a vacuum dryer with an ejector system does not exist. The main disadvantage of such a dryer with an ejector system would be limited capacity due to the impossibility of continuous production and the construction problems of maintaining a vacuum in large vessels; thus, it could not be used for mass production. Accordingly, the goals of this research could be separated into three parts. The first part was to design, construct and install a new prototype of a vacuum dryer by using an ejector system instead of a vacuum pump used in convectional vacuum driers. The second was to test this new prototype, analyze the quality of the obtained dried fruit and compare it to that of dried fruit obtained in a convectional vacuum dryer with a vacuum pump. Finally, the third was to present a theoretical calculation of energy efficiency and compare the energy consumption of the prototype to that of industrial dryers. Regarding the highly valuable nutritional and sensory properties of sour cherry and apricot, their good drying characteristics, and also their good properties in dried form, they were chosen as samples in this research to compare their properties when dried in a vacuum dryer with a vacuum pump to those when dried in vacuum driers with ejector systems.

## 2. Materials and Methods

### 2.1. Samples

Sour cherries and apricots were purchased at the local market (2020). The samples were at the stage of technological maturity and quality. Fruits were pitted, and the samples were frozen and stored at −20 °C until drying. Prior to freezing, the apricots were cut into slices of 3 mm thickness, while the sour cherries were frozen as whole fruit.

### 2.2. Reagents

The following reagents were used in the experimental work: Gallic acid—Sigma-Aldrich GmbH, Steinheim, Germany; Folin-Ciocalteu reagent—Sigma-Aldrich GmbH, Steinheim, Germany; (±)-Catehin—Sigma-Aldrich GmbH, Steinheim, Germany; 2,4,6-Tris (2-pyridyl) -s-triazine (TPTZ)—Sigma-Aldrich GmbH, Steinheim, Germany; 2,2-Diphenyl-2-picryl hydrazyl hydrate (DPPH)—Sigma-Aldrich GmbH, Steinheim, Germany; 2,2′-Azino-bis (3-ethylbenzothiazoline-6-sulfonic acid) diammonium salt (ABTS)—Sigma-Aldrich GmbH, Steinheim, Germany; Trolox (6-hydroxy-2,5,7,8-tetramethylchroman-2-carboxylic acid)—Sigma-Aldrich GmbH, Milan, Italy. All other chemicals and reagents used in the experimental work were of analytical purity.

### 2.3. Drying Procedures

#### 2.3.1. Vacuum Dryer with an Ejector System: Designing of an Innovative Vacuum Dryer with an Ejector System

Innovation in the application of the ejector system on the vacuum dryer was successfully realized within the innovation project “Development of prototype of vacuum drier with the ejector system” (No. 1068) within the Technology Transfer Program, Innovation Found of the Republic of Serbia. The prototype of the innovative vacuum dryer with an ejector system was designed, constructed, installed and tested in the laboratory of the research group for Technology of fruit and vegetable products of the Faculty of Technology Novi Sad, University of Novi Sad following the global stepwise approach shown in Table 1.

#### 2.3.2. Vacuum Dryer with a Vacuum Pump

A vacuum dryer with a vacuum pump is constructed and installed at the Faculty of Technology Novi Sad, University of Novi Sad (Serbia). A detailed description of the vacuum dryer equipment and vacuum drying process is presented in a technical solution [22] and also published by Šumić et al. [23]. This dryer consists of a cylindrical chamber made of steel with a volume of approximately 70 L, a vacuum pump that ensures a pressure in the chamber of 2 mbar and a condenser. The aluminum heating plate is fixed to a special frame inside the chamber. The vacuum dryer with a vacuum pump is equipped with a PLC control unit that registers all operating parameters: pressure in the vacuum chamber, temperature of the heating plate, change in the mass of the raw material being dried and the temperature of the condenser during the process of vacuum drying. In this way, the system controls the level of electricity supplied to the heaters to ensure a temperature not higher than 75 °C. The most important technical characteristics related to the device are as follows: operating temperature range from 25 to 75 °C; temperature sensor sensitivity ±0.3 °C; maximum load 1000 g; mass measurement precision 0.1 g; working pressure range from 2 to 1000 mbar; sensitivity of the pressure sensor ±0.5 mbar. During drying, the samples were evenly distributed on the hot plate in a thin layer. Weight loss was recorded at 5 min intervals, and drying was continued until no mass change was detected (final moisture content in equilibrium), when the drying was ended.

#### 2.3.3. Experimental Design

The initial planning of the experiment predicted the drying of sour cherries and apricots in a vacuum dryer (VD) with a vacuum pump (P) and in the prototype of an innovative vacuum dryer with an ejector system (E) at different temperatures and the lowest values of pressure that each dryer could achieve. Samples of sour cherries and apricots were dried at different temperatures of 40, 50, 60, 70 and 80 °C (SC1, SC2, SC3, SC4, SC5 and A1, A2, A3, A4, A5, respectively) in both dryers until a constant mass was achieved. The following physical, chemical and biological properties were analyzed in all dried samples: moisture content, a_w_ value, total phenol and flavonoid content and antioxidant activity (FRAP, DPPH and ABTS assay). Total monomeric anthocyanin content was also analyzed in dried sour cherry samples. The vacuum drying conditions of sour cherries and apricots are presented in Table 2. The results of analyses of dried sour cherry samples are shown in Table 3, Table 4 and Table 5, while the results of analyses of dried apricot samples are shown in Table 6 and Table 7.

It is important to mention how the end point for drying was reached for the drying processes, as it has previously been explained in papers by Sun et al. [24], Li et al. [25] and Deng et al. [26]. Drying in a vacuum dryer with a vacuum pump was conducted first, and in this dryer, drying was continued until no mass change was detected, which is explained in detail in Section 2.3.2. Based on this recorded time obtained for each drying condition and for each fruit separately, the drying in the vacuum dryer with the ejector system was conducted in the same manner, i.e., for the same duration. These conditions were chosen in order to obtain better conclusions since, this way, all drying conditions in both dryers were the same.

The prototype is certified by the authorized designer, and in the framework of the project, a risk assessment for safety and health at work was carried out, which reached the conclusion that the device fulfills all requirements for safe work.

### 2.4. Analyses

#### 2.4.1. Moisture Content

Moisture content (MC) was determined by drying samples at 105 °C until a constant weight was reached.

#### 2.4.2. Water Activity

Water activity (a_w_) was determined by placing approximately 2.5 g of chopped sample into the plastic measuring container of the measuring cell with the probe inside the a_w_-meter (LabSwift, Novasina, Switzerland). After reaching equilibrium humidity, the a_w_ value is read out on a_w_-meter display.

#### 2.4.3. Texture Analysis

Instrumental texture measurements were performed using a Texture Analyzer (TE32; Stable Micro Systems, Godalming, Surrey, UK). The shear force (SF) of dried samples was measured using a knife blade. TA settings were the following: test speed—1.0 mm/s and load cell—5 kg. Shear force is expressed as force (g) required to cut the sample. Penetration force (PF) of dried samples was measured using a 2 mm stainless cylindrical probe. TA settings were the following: test speed—2.0 mm/s and load cell—5 kg. The sample was placed centrally in relation to the cylindrical probe. Penetration force is expressed as the force (g) required to penetrate through the samples.

#### 2.4.4. Total Phenolic, Flavonoid, Anthocyanin Content and Antioxidant Analysis

Total phenolic content (TPC) was determined by the Folin–Ciocalteu colorimetric assay [27]. Gallic acid was used as a standard, while sample absorbance was measured at 750 nm. Results are expressed as mg of gallic acid equivalents per 100 g of dried weight (mg GAE/100 g DW). Furthermore, aluminum chloride coloimetric assay [28] was used for total flavonoid content (TFC) determination. Catechin was used as a standard, while sample absorbance was measured at 510 nm. Results are expressed as mg of catechin equivalents per 100 g of dried weight (mg CE/100 g DW). Total monomeric anthocyanin (TMAC) content was obtained by the Giusti and Wrolstad [29] method, which is based on the pH differential method described by Fuleki and Francis [30]. Results are expressed as mg of cyanidin-3-glucoside equivalents of dried weight (mg CGE/100 g DW). The FRAP assay was conducted using a slightly modified method first presented by Benzie and Strain [31]. Results are expressed as the mg equivalent of Fe^2+^ ions per g of dry matter (mg Fe^2+^/g DW). The DPPH assay was measured using a modified method originally presented by Brand-Williams et al. [32]. Results are expressed as mg Trolox equivalents per g of dry weight (mg Trolox/g DW). The ABTS test was measured using a modified method originally described by Re et al. [33]. Results are expressed as mg Trolox equivalents per g of dry weight (mg Trolox/g DW). A detailed description of the analysis (moisture content, water activity, and total phenolic, flavonoid, and monomeric anthocyanin content) is shown in detail by Tepić Horecki et al. [34], while a detailed description of antioxidant activity assays is presented by Vakula et al. [35].

### 2.5. Statistical Data Analysis

All experiments were performed in triplicate for statistical purposes, except texture analysis, where measurements were repeated five times. Results were presented as mean values and standard deviations (SD). The differences between the results obtained with vacuum drying with a vacuum pump and vacuum drying with an ejector system were analyzed by univariate analysis of variance (ANOVA, *p* < 0.05) in order to differentiate the samples by using Tukey’s multiple comparison test and a 0.05 criterion. The program Minitab 16 Trial was used for the ANOVA.

## 3. Results and Discussion

### 3.1. Prototype of an Innovative Vacuum Dryer with an Ejector System

**Figure 1:**
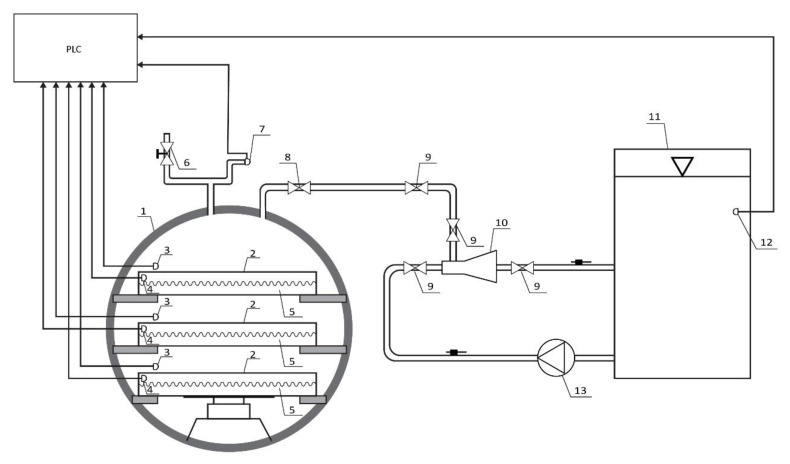
Schematic diagram of a prototype of an innovative vacuum dryer with an ejector system: 1. chamber; 2. heating plate; 3. sample temperature sensor; 4. heating plate temperature sensor; 5. heater; 6. vent valve; 7. pressure measuring sensor; 8. non-return valve; 9. locking element; 10. ejector; 11. cooled water tank; 12. sensor for measuring water temperature in the tank; 13. centrifugal pump.

**Figure 2:**
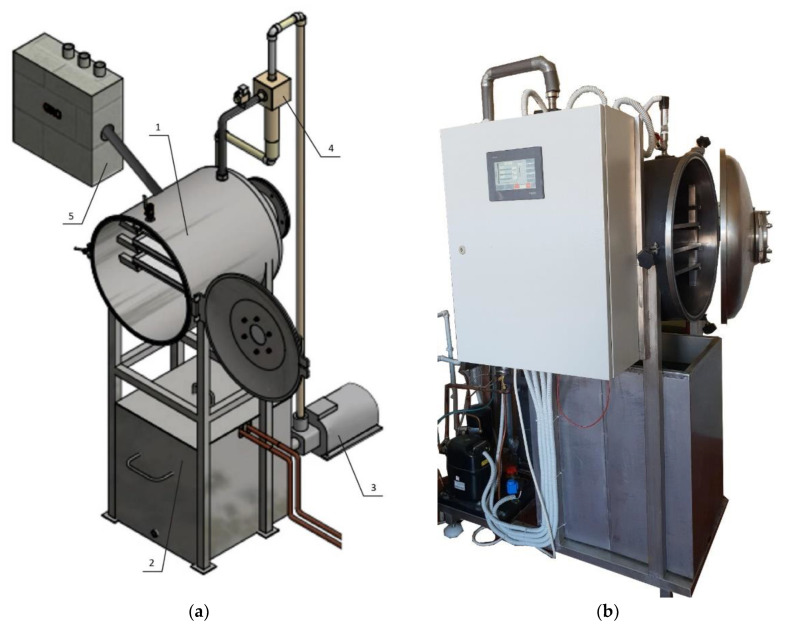
3D view of a prototype of an innovative vacuum dryer with an ejector system: 1. chamber; 2. cooled water tank; 3. centrifugal pump; 4. ejector; 5. PLC control unit (**a**); Prototype of an innovative vacuum dryer with an ejector system installed in the laboratory (**b**).

The prototype of an innovative vacuum dryer with an ejector system consists of a cylindrical chamber and an ejector system (Figure 1 and Figure 2). The chamber, dimensions of φ600 mm and a wall thickness of 4 mm, is made of stainless steel. The chamber door is made of the same material with dimensions of φ600 mm and has embedded glass with dimensions of φ100 mm in order to facilitate visual control of the drying process. The drying chamber is equipped with heating plates with dimensions 500 × 400 mm made of technical aluminum with embedded heaters with a power of 200 W, which enable the heating of material up to 80 °C.

The ejector system consists of an ejector, a centrifugal pump, a pipe and a water cooling tank. The four-stage centrifugal pump (model CR5-12, manufactured by Grundfos from Denmark) could achieve an overpressure of 0.572 MPa (5.72 bar), which allows the flow of water in the ejector to be around 10 m^3^/h. The tank contains 120 L of cold water up to 5 °C. During drying, the water in the tank is cooled by a tubular heat exchanger that is built into the tank and through which the cooling fluid (freon) flows.

The prototype of the innovative vacuum dryer with an ejector system is equipped with a programmable logic controller (PLC), which enables automatic control of the drying process. The PLC has the ability to measure and memorize absolute pressure, tank water temperature, temperature of heat plates and temperature of samples. Pressure is measured using a pressure measuring sensor (model GRQ000A00, Georgin, France), tank water temperature using a temperature measuring sensor (model TS-01_PT100, Nigos, Nis, Serbia), temperature of hot plates and temperature of a sample dried using a temperature measuring sensor (model TS-03_PT100, Nigos). The ejector system and the heater are controlled by the PLC, so that an absolute pressure of 20 mbar could be achieved in the working chamber and the set value of the heating plate temperature could be maintained during drying.

The most important technical characteristics of the device are the operating temperature range of 20 to 80 °C, the operating pressure range of 20–1000 mbar, the maximum mass of the drying sample of 5 kg, the sensitivity of the temperature measuring sensor ±0.3 °C, and the sensitivity of the pressure measuring sensor ±0.5 mbar.

A centrifugal pump takes water from the pre-cooled water tank to a temperature of 5 °C and directs it through the pipe into the ejector. The water goes through the ejector, i.e., through the venturi, whereby it gains acceleration and, according to Bernoulli’s law, reduces the pressure in the chamber in which the sample is dried at a given temperature. By reducing the pressure in the chamber, i.e., by achieving a vacuum, the evaporation of water from the dried raw material is accelerated. The released water vapor is removed by an ejector by mixing the vapor with water, which flows through the ejector. The mixture of water and steam enters through a pipe into the cooled water tank, where the steam condenses. The centrifugal pump constantly takes water from the tank, and thus the drying cycle takes place continuously. The temperature of the water in the tank must not exceed 5 °C in order to ensure constant operating conditions for the ejector system, as the vacuum is disturbed when the temperature of the cooling fluid increases.

The main advantages of the prototype of the innovative vacuum dryer with an ejector system compared to the vacuum dryer with a vacuum pump are:

- Significantly less investment in equipment, as there is no need for a condenser and a vacuum pump to achieve a vacuum. An ejector system has been applied, which requires significantly less investment in terms of equipment.
- Significantly lower equipment maintenance costs since the ejector system has no moving parts.

The main disadvantage of this prototype is the limited capacity of the device, so it cannot be used for mass production but is primarily intended for the production of small quantities of top-quality dried fruit. The capacity of the dryer is limited due to the impossibility of continuous production and the construction problems of maintaining a vacuum in large vessels.

**Figure 3:**
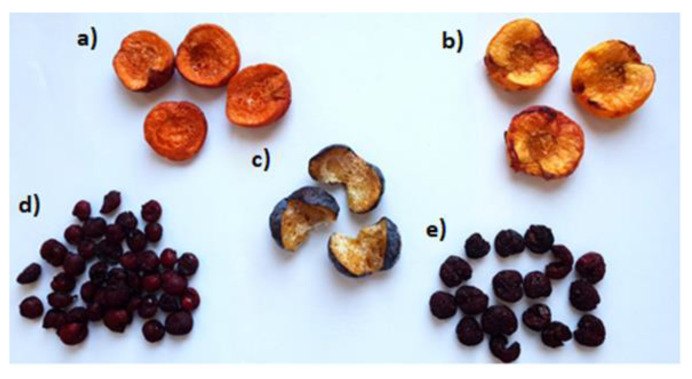
The samples of stone fruit dried with the vacuum dryer with an ejector system: (**a**) apricot; (**b**) peach; (**c**) plum; (**d**) sour cherry; (**e**) sweet cherry.

The prototype of the innovative vacuum dryer with an ejector system is shown in Figure 2, and samples of stone fruit dried with the vacuum dryer with an ejector system are shown in Figure 3.

### 3.2. Comparative Analysis of the Physical, Chemical and Biological Properties of Fruit Dried Using a Vacuum Dryer with a Vacuum Pump or an Ejector System

Sour cherries have been recognized as excellent sources of numerous healthy ingredients, such as phenolic compounds and particularly significant anthocyanin content. Phenol carboxylic acids, flavanols and their derivatives are the most abundant antioxidant compounds present in sour cherries [36]. Besides providing the dark red color of fruits, anthocyanins possess strong antioxidant capacity and, accordingly, may achieve significant health benefits [37,38]. The quite rich content of the mentioned biologically active compounds with a wide range of significant functional properties, together with tempting sensory attributes, make sour cherries one of the most interesting and studied raw materials in recent years [39]. Numerous suitable kinds of processing and preservation methods could be applied in order to maintain the appropriate quality characteristics of various seasonal stone fruits and allow their availability on the market throughout the year [12]. As a fruit species mostly intended for industrial production, sour cherries are rarely consumed fresh, so the vast majority are processed into a wide range of products, such as juice, jelly, jam, marmalade, puree and alcoholic drinks. They can be dried, canned, frozen or used as an ingredient in the production of confectionery items [40,41,42]. Raw sour cherry samples were analyzed, and the following results were obtained: MC 84.10%; a_w_ 0.930; SF 249 g; PF 98 g; TPC 2812 mg GAE/100 g DW; TFC 2364 mg CE/100 g DW; TMAC 903 mg CGE/100 g DW; FRAP 28.22 mg Fe^2+^/g DW; DPPH 40.02 mg Trolox/g DW; ABTS 112.00 mg Trolox/g DW.

#### 3.2.1. Physical Characteristics of Dried Sour Cherry Samples

All physical characteristics of dried sour cherry samples are presented in Table 3. The moisture content in sour cherry samples dried at higher applied temperatures (60, 70 and 80 °C) was higher in samples dried in a vacuum dryer with a vacuum pump (VD-P) compared to samples dried in a prototype of an innovative vacuum dryer with an ejector system (VD-E). In sour cherry samples dried at lower applied temperatures (40 and 50 °C), moisture content was higher in samples dried in VD-E at the level of statistical significance (*p* < 0.05). Based on the results of moisture content obtained in this study, it was noticed that all dried sour cherry samples in both VD-P and VD-E meet the requirements of quality and safety according to which dried fruit must not contain more than 27% water (Official Gazette of SFRY, 1/79) [43], which was very important to see the similarities of the samples dried in both dryers.

According to the obtained a_w_ values, it was noted that in the samples of sour cherries dried at all applied temperatures, a higher a_w_ value was measured in the samples dried in VD-P compared to the samples dried in VD-E. These results indicate that, in terms of the investigated parameter, the a_w_ value, the prototype of the innovative vacuum dryer with an ejector system gave better results compared to the vacuum dryer with a vacuum pump. It was noticed that in the case of dried sour cherry samples, all investigated samples met the requirements of quality and safety in terms of a_w_ values.

Based on the obtained results of texture analysis, i.e., values for share and penetration force, it was noticed that the texture of sour cherry samples dried in VD-P and VD-E did not differ at the level of statistical significance (*p* < 0.05) for any value of investigated temperature, which indicates that in terms of the texture of the dried samples, the prototype of the innovative vacuum dryer with an ejector system resulted in the same good results as the vacuum dryer with a vacuum pump.

#### 3.2.2. Chemical and Biological Characteristics of Dried Sour Cherry Samples

All the chemical and biological properties of dried sour cherry samples are presented in Table 4 and Table 5. Regarding the content of bioactive components in sour cherry dried samples, in all samples dried in VD-E, a higher content of total phenols was observed, while in terms of the content of total flavonoids and monomeric anthocyanins, better results were achieved, i.e., higher values of these components were obtained in samples of sour cherries dried in VD-P. In accordance with other results, the obtained results of antioxidant activity determined by FRAP, DPPH and ABTS assays indicate that the samples of sour cherries dried in VD-P and VD-E possess similar and satisfactory biological properties.

Unlike sour cherries, apricot fruits possess highly balanced sweetness and acidity induced by the content of sugars and organic acids, respectively, which contribute to their aromatic and rich taste [44]. This stone fruit is distinguished by different shades of yellow and orange, which are derived from carotenoids, pigments naturally occurring in numerous fruits and vegetables [45,46]. Within the last decade, apricots have become a raw material of great interest in the scientific research field thanks to the presence of important beneficial nutrients and molecules with different bioactivities in humans, such as polyphenols, β-carotene, organic acids, mineral elements, and vitamins [14,21]. Apricots can be consumed fresh, dried or as an excellent starting raw material for processing into jams, jellies, juices, nectars and pulp [47]. Due to the short shelf life of fresh apricots and the rapid ripening and softening of fruits, the majority of cultivated apricots have been consumed as dried fruits [26]. Dried fruits represent relative concentrated forms of fresh fruits [48], and in accordance with that, high-quality dried fruit completely requires fresh raw material with outstanding initial physico-chemical properties [41]. Nowadays, consumer demands in terms of dried fruits, including apricots, are related to maintaining their rich nutritional value as much as possible and also ensuring high product safety during their shelf life [49]. Generally, dried fruits are considered safe from the aspect of microbial contamination, especially in conditions of low water activity where the potential for food pathogen growth is limited [50]. Raw apricot samples were analyzed, and the following results were obtained: MC 88.94%; a_w_ 0.927; SF 12.3 g; PF 212 g; TPC 327 mg GAE/100 g DW; TFC 471 mg CE/100 g DW; FRAP 2.9 mg Fe^2+^/g DW; DPPH 9.73 mg Trolox/g DW; ABTS 9.01 mg Trolox/g DW.

#### 3.2.3. Physical Characteristics of Dried Apricot Samples

The physical characteristics of dried apricot samples are presented in Table 6. Moisture content in apricot samples dried at lower applied temperatures (50 and 60 °C) was higher in the samples dried in a vacuum dryer with a vacuum pump (VD-P) compared to samples dried in a prototype of an innovative vacuum dryer with an ejector system (E). In apricot samples dried at higher temperatures, moisture content was higher in samples dried in VD-E, but not at the level of statistical significance (*p* < 0.05). In the study of Hussain and Yasmin [51], a moisture content range between 10.61 and 15.10% was recorded in the investigated samples of different varieties of dried apricots. In addition, Witherspoon and Jackson [52] showed moisture content in dried apricots between 10 and 25%. As in the case of sour cherries, the same was true of apricots: Based on the results of moisture content in this study, it was noticed that all samples of dried apricots in both VD-P and VD-E meet the quality and safety requirements for dried fruit, which should not contain more than 27% water [43].

As in the case of moisture content, the a_w_ value of apricot samples dried at lower temperatures (50 and 60 °C) was higher in samples dried in VD-P compared to samples dried in VD-E. In addition, the a_w_ value of apricot samples dried at the highest applied temperature was higher in samples of apricots dried in VD-E compared to samples dried in VD-P. In apricot samples dried at 60 and 70 °C, the a_w_ value of the samples dried in VF-E was higher compared to the samples dried in VD-P, with no statistically significant difference between the samples dried at 70 °C. Considering that convectively dried fruit should be stored in the range of a_w_ values between 0.45 and 0.54, and lyophilized fruit between 0.46 and 0.63 [6], it was concluded that all dried apricot samples investigated in this research meet these quality and safety requirements.

In addition, as in the case of dried sour cherry samples, it was noted that the texture (share and penetration force) of apricot samples dried in VD-P and VD-E did not differ at the level of statistical significance (*p* < 0.05) at all investigated drying temperatures (40–80 °C).

#### 3.2.4. Chemical and Biological Characteristics of Dried Apricot Samples

The chemical and biological properties of dried apricot samples are presented in Table 6. Regarding the content of bioactive components and antioxidant activity of dried apricot samples, it was noted that samples dried in VD-E at higher applied temperatures (60, 70 and 80 °C) possess a higher content of total phenols and higher antioxidant activity determined by FRAP and DPPH tests compared to samples dried in VD-P. In the apricot samples dried in VD-P, a higher content of flavonoids was obtained in all analyzed samples compared to samples of apricots dried in VD-E under the same conditions of temperature, pressure and time. A comparison of apricot samples dried at 40 and 60 °C in VD-P and VD-E did not show a significant difference (*p* < 0.05) in the content of total flavonoids. The content of antioxidant activity determined by the DPPH assay in apricot samples dried at a temperature of 80 °C was higher in samples dried in VD-P, but not at the level of statistical significance (*p* < 0.05). Based on the antioxidant ABTS assay, apricot samples dried in VD-E at 60 °C had higher antioxidant activity compared to samples dried in VD-P, while in the case of other samples, higher ABTS assay contents were recorded in samples dried in VD-P. There was no significant difference (*p* < 0.05) between the samples dried in VD-P and VD-E at 40 °C based on the antioxidant DPPH assay. Igual et al. [53] noticed a total phenol content of 64.73; 64.90; 60.00; 81.00 mg GAE/100 g fresh sample in hot air dried apricot samples at 40 °C, in hot air dried apricot samples at 60 °C; in hot air in combination with microwave drying and microwave drying, respectively.

### 3.3. Calculation of Energy Efficiency of Vacuum Dryer with an Ejector System

The vacuum dryer with the ejector system has three heating plates on which the fruit for drying is placed. The dryer can hold up to 15 kg of raw fruit. The vacuum is generated by a water jet ejector. The driving fluid is water. The water receives pressure from a multistage centrifugal pump. The drying process takes from 8 to 10 h.

According to the following compositions of the equations [54,55], the energy efficiency of a vacuum dryer with an ejector system was calculated:

The required electrical power:

(1) $$\Phi_{e}=P\cdot t=2090\;W\times 10\;h=20,900\;Wh=20.9\;kWh$$

where:

t—10 h, duration of the drying process under vacuum.
*P*—2090 W, pump power, calculated as following:

(2) $$P=\frac{Volumetric\;Flow\;rate\cdot Pressure}{Total\;Efficiency}=\frac{\frac{10}{3600}\frac{m^{3}}{s}\times 57,200\;Pa}{0.76\;(frequent\;value\;in\;centrifugal\;pumps)}=2090\;W$$

The amount of dried fruit:

(3) $$G_{dm}=G_{1}\times \frac{100-w_{1}}{100-w_{dm}}=15\times \frac{100-80}{100-20}=3.75\;kg$$

where:

*G*_1_—15 kg, the amount of raw fruit in the chamber;
*G_dm_*—the amount of dried fruit (kg);
*w*_1_—80% relative water content in raw fruit (%);
*w_dm_*—20% relative water content in dried fruit (%).

The amount of water to be evaporated:

(4) $$G_{w}=G_{1}-G_{dm}=15-3.75=11.25\;kg$$

where:

*G*_1_—15 kg the amount of raw fruit in the chamber;
*G_dm_*—3.75 kg the amount of dried fruit.

Required thermal power:

(5) $$\Phi =\Phi_{h}+\Phi_{hl}=\frac{29,332\times 1000}{10\times 3600}+523=814.8+523=1338\;W$$

where:

Φ*_h_*—heating energy;
Φ*_hl_*—heat loss energy.

Mass heating:

(6) $$E_{h}=E_{dm}+E_{wv}=330+29,002=29,332\;kJ$$

Heat of dry mass heating:

(7) $$E_{dm}=G_{dm}\times c\times 40{}^{\circ}=3.75\times 2.2\times 40=330\;\mathrm{kJ}$$

where:

*c*-between 2 and 2.5 kJ/kg °C [56], the heat capacity of the dried mass (for dried fruit, i.e., apricot, it is on average higher).

Heat of water vapor evaporation:

(8) $$E_{wv}=\left(G_{1}-G_{dm}\right)\times 2578=11.25\times 2578=29,002\;kJ$$

Carrying out the average based on the masses of water to be evaporated when the moisture is higher than Wc and when it is lower than Wc, an average thermal energy value of 2578 kJ is obtained if the final moisture W_2_ is 20% [57,58,59].

Heat losses:

(9) $$\Phi_{hl}=U\times A\;*\;40=1.09\;*\;12\;*\;40=523\;W$$

(10) $$U=\frac{1}{\frac{1}{he}+\frac{s}{\lambda }+\frac{1}{hi}}=\frac{1}{\frac{1}{4}+\frac{0.004}{16}+\frac{1}{1.49}}=1.09\;\frac{W}{m^{2}}K$$

where:

*σ*—5.67 W/m^2^K^4^, the heat transfer coefficient from the absolute black body to the environment;
*λ*—16 W/mK, thermal conductivity of stainless steel
s—4 mm thickness of the chamber wall
hi = he × 0.05 ^ 0.33 = he × 0.372 = 1.49
he = 4 W/m^2^K

Total thermal energy to evaporate 11.25 kg of water:

(11) $$E_{t}=1338\times 10=13.38\;kWh=13.38\times 3600=48,168\;kJ$$

Total thermal energy per kg of evaporated water:

(12) $$e_{t}=\frac{E_{t}}{G_{1}-G_{dm}}=\frac{48,168}{11.25}=4282\;\frac{kJ}{kg}of\;water$$

Comparison with other types of dryers:

A similar value of the total thermal energy is also present, for example, in Perry (1984) [60], where an average thermal efficiency of 60% is indicated, i.e., a total thermal energy of 2547/0.6 = 4254 kJ per kg of evaporated water. The adjective total is indicated because it must also include the heat losses and the greater thermal energy required by the bound water. The result of this comparison confirms that there is no difference between the use of the ejector and the use of a vacuum pump, for example, a liquid-ring pump. On the other hand, the thermal energy required for evaporation is the same in the two cases because the apricot is the same, and the heat losses are also the same given that the use of the same drying chamber is assumed.

## 4. Conclusions

The main result of this research was achieved by designing, constructing, installing and testing the innovative prototype of a vacuum dryer with an ejector system in the laboratory of the Technology of fruit and vegetable products of the Faculty of Technology Novi Sad, University of Novi Sad. Analyzing and comparing the moisture content, a_w_ value, share and penetration force, total phenol, flavonoid and anthocyanin content and antioxidant activity (FRAP, DPPH and ABTS test) of sour cherries and apricots dried in a vacuum dryer with a vacuum pump and vacuum dryer with an ejector system, it was concluded that vacuum dryers with ejector systems yield similar results as those obtained using vacuum dryers with vacuum pumps in terms of all tested physical, chemical and biological properties of dried samples. This was concluded based on the similarities of the results of some of the most important parameters of the product safety and quality, such as the a_w_ value and total phenol content, respectively. In dried sour cherry, the a_w_ values ranged from 0.250 to 0.521 with the vacuum pump and from 0.232 to 0.417 with the ejector system; the total phenol content ranged from 2322 to 2765 mg GAE/100 g DW with the vacuum pump and from 2327 to 2617 mg GAE/100 g DW with the ejector system. In dried apricot, the a_w_ ranged from 0.176 to 0.405 with the vacuum pump and from 0.166 to 0.313 with the ejector system; total phenol content ranged from 392 to 439 mg GAE/100 g DW with the vacuum pump and from 378 to 428 mg GAE/100 g DW with the ejector system. Taking into account all advantages of a vacuum dryer with an ejector system, in terms of less investment in equipment and lower maintenance costs, and the results obtained by using the innovative dryer to test the physical, chemical and biological properties of dried samples, a conclusion was drawn based on all the criteria in terms of process economy and quality of the dried product, and it can be stated that the innovative vacuum dryer presents a good choice of fruit drying technique. Quality criteria in terms of physical, chemical, and biological properties of dried fruit are met in the vacuum dryer with a vacuum pump and in the case of the dryer with an ejector system, while in the economic sense of investment and maintenance, the innovative dryer with an ejector system stands out. Furthermore, another conclusion regarding energy efficiency in this research was that there is no difference between the use of an ejector and the use of a vacuum pump.

## Figures and Tables

**Table 1:** Project task of making a prototype of a laboratory vacuum dryer with an ejector system.

No	Prototype Parts	Description
1	Working chamber	Construct a working chamber with a door.
2	Plates	Inside the chamber, install three heating plates with heaters with a total usable area of 0.6 m^2^ and a total load mass of 5 kg/m^2^.
3	Ejector system	Install ejector, centrifugal pump, water tank and cooler.
4	Sensors	Install pressure (±0.5 mbar) and temperature (±0.3 °C) sensors.
5	Control unit	Enable automatic process control using a PLC (Programmable Logic Controller) control unit.
6	Software	Install and adjust the process control software using the PLC control unit, the input parameters that are controlled are the pressure in the chamber and the temperature of the heating plates. Enable recording of process time, chamber pressure and heating plate temperature during the process.
	Working parameters	
1	Heating plateau temperature	Allow the plateau to heat up to a temperature between 40 and 80 °C and maintain the plateau temperature during the process, with a homogeneous temperature field over the entire surface of the plateau.
2	Chamber pressure	Provide a minimum working pressure in the chamber of 50 mbar or lower, reaching the working pressure within a maximum of 15 min from the start of the process and maintaining the working pressure during the process.
3	Water temperature in the tank	Allow the water in the ejector system tank to cool to a temperature between 0 and 5 °C and maintain the water temperature during the process.
	Other conditions	
1	Noise	The maximum noise generated by the prototype must not exceed 40 dB.
2	Construction materials	Materials in contact with food must be suitable for the food industry.

**Table 2:** Vacuum drying conditions of sour cherry and apricot dried in a vacuum dryer with a vacuum pump (P) and in a vacuum dryer with an ejector system (E).

Sample	T (°C)	p (mbar)	t (h)
SC1-P	40	20	5.3
SC1-E	40	20	5.3
SC2-P	50	20	5.1
SC2-E	50	20	5.1
SC3-P	60	20	4.0
SC3-E	60	20	4.0
SC4-P	70	20	3.4
SC4-E	70	20	3.4
SC5-P	80	20	3.8
SC5-E	80	20	3.8
A1-P	40	20	9.7
A1-E	40	20	9.7
A2-P	50	20	8.6
A2-E	50	20	8.6
A3-P	60	20	8.3
A3-E	60	20	8.3
A4-P	70	20	6.9
A4-E	70	20	6.9
A5-P	80	20	6.2
A5-E	80	20	6.2

**Table 3:** Experimentally obtained values of moisture content, a_w_ values and texture analysis (share force and penetration force) in dried sour cherry samples dried using VD-P and VD-E.

*	MC		a_w_		SF		PF	
	VD-P	VD-E	VD-P	VD-E	VD-P	VD-E	VD-P	VD-E
SC1	19.78 ± 0.25 ^b^	22.91 ± 0.52 ^a^	0.521 ± 0.000 ^a^	0.417 ± 0.001 ^b^	3.9 ± 0.6 ^a^	3.4 ± 0.6 ^a^	0.8 ± 0.2 ^a^	1.0 ± 0.4 ^a^
SC2	14.95 ± 0.23 ^b^	17.20 ± 0.28 ^a^	0.381 ± 0.001 ^a^	0.362 ± 0.000 ^b^	3.8 ± 0.8 ^a^	5.0 ± 0.5 ^a^	0.9 ± 0.3 ^a^	1.1 ± 0.3 ^a^
SC3	14.35 ± 0.57 ^a^	12.70 ± 0.43 ^b^	0.285 ± 0.002 ^a^	0.258 ± 0.002 ^b^	5.1 ± 0.5 ^a^	5.4 ± 0.3 ^a^	1.2 ± 0.6 ^a^	1.3 ± 0.4 ^a^
SC4	14.37 ± 0.18 ^a^	12.61 ± 0.47 ^b^	0.289 ± 0.007 ^a^	0.259 ± 0.001 ^b^	5.3 ± 0.2 ^a^	4.6 ± 0.5 ^a^	1.0 ± 0.2 ^a^	1.0 ± 0.1 ^a^
SC5	11.61 ± 0.26 ^a^	10.40 ± 0.32 ^b^	0.250 ± 0.001 ^a^	0.232 ± 0.000 ^b^	4.6 ± 0.7 ^a^	4.3 ± 0.9 ^a^	1.0 ± 0.1 ^a^	0.9 ± 0.1 ^a^

* MC—moisture content (%); a_w_—water activity; SF-share force (kg); PF—penetration force (kg); SC-sour cherry; VD-P—vacuum dryer with a vacuum pump; VD-E—vacuum dryer with an ejector system; Means that do not share a letter are significantly different (*p* < 0.05); Letters ^a^ and ^b^—differences between the sample dried at the same conditions but with a different vacuum dryer (vacuum pump (VD-P) and ejector system (VD-E)).

**Table 4:** Experimentally obtained values of total phenolic, flavonoid and monomeric anthocyanin content in sour cherry samples dried using VD-P and VD-E.

*	TPC		TFC		TMAC	
	VD-P	VD-E	VD-P	VD-E	VD-P	VD-E
SC1	2528 ± 0.01 ^b^	2617 ± 0.04 ^a^	2063 ± 10 ^a^	2034 ± 21 ^b^	697 ± 0.004 ^b^	710 ± 0.003 ^a^
SC2	2317 ± 0.02 ^b^	2377 ± 0.02 ^a^	2051 ± 18 ^a^	1935 ± 1 ^b^	830 ± 0.008 ^a^	736 ± 0.005 ^b^
SC3	2765 ± 0.03 ^a^	2601 ± 0.03 ^b^	2254 ± 27 ^a^	2058 ± 16 ^b^	834 ± 0.01 ^b^	892 ± 0.005 ^a^
SC4	2322 ± 0.03 ^a^	2327 ± 0.03 ^a^	2254 ± 06 ^a^	2014 ± 18 ^b^	823 ± 0.007 ^a^	693 ± 0.006 ^b^
SC5	2495 ± 0.01 ^b^	2552 ± 0.01 ^a^	2020 ± 13 ^a^	1903 ± 9 ^b^	810 ± 0.008 ^a^	791 ± 0.004 ^b^

* TPC—total phenolic content ( ); TFC—total flavonoid content (mg CE/100 g DW); TMAC—total monomeric anthocyanin content (mg CGE/100 g DW); SC—sour cherry; VD-P—vacuum dryer with a vacuum pump; VD-E—vacuum dryer with an ejector system; Means that do not share a letter are significantly different (*p* < 0.05); Letters ^a^ and ^b^—differences between the sample dried at the same conditions but with a different vacuum dryer (vacuum pump (VD-P) and ejector system (VD-E)).

**Table 5:** Experimentally obtained values of antioxidant activity (FRAP, DPPH and ABTS assay) in sour cherry samples dried using VD-P and VD-E.

*	FRAP		DPPH		ABTS	
	VD-P	VD-E	VD-P	VD-E	VD-P	VD-E
SC1	18.16 ± 0.15 ^b^	20.08 ± 0.21 ^a^	18.49 ± 0.10 ^b^	19.19 ± 0.22 ^a^	95.47 ± 0.4 ^b^	99.47 ± 0.8 ^a^
SC2	16.43 ± 0.04 ^b^	17.06 ± 0.05 ^a^	18.65 ± 0.05 ^b^	19.12 ± 0.05 ^a^	83.89 ± 1.0 ^a^	85.75 ± 0.6 ^a^
SC3	16.30 ± 0.09 ^b^	16.60 ± 0.08 ^a^	26.70 ± 0.09 ^a^	23.27 ± 0.16 ^b^	106.14 ± 0.7 ^a^	92.23 ± 0.7 ^b^
SC4	18.77 ± 0.14 ^a^	17.34 ± 0.18 ^b^	28.47 ± 0.24 ^a^	28.25 ± 0.28 ^a^	98.72 ± 0.2 ^a^	95.34 ± 0.8 ^b^
SC5	18.32 ± 0.03 ^a^	16.27 ± 0.07 ^b^	28.24 ± 0.31 ^a^	25.97 ± 0.19 ^b^	100.88 ± 1.4 ^a^	95.91 ± 1.0 ^b^

* FRAP assay (mg Fe^2+^/g DW); DPPH assay (mg Trolox/g DW); ABTS assay (mg Trolox/g DW); SC-sour cherry; VD-P—vacuum dryer with a vacuum pump; VD-E—vacuum dryer with an ejector system; Means that do not share a letter are significantly different (*p* < 0.05); Letters ^a^ and ^b^—differences between the sample dried at the same conditions but with a different vacuum dryer (vacuum pump (VD-P) and ejector system (VD-E)).

**Table 6:** Experimentally obtained values of moisture content, a_w_ values and texture analysis (share force and penetration force) in dried apricot samples dried using VD-P and VD-E.

*	MC		a_w_		SF		PF	
	VD-P	VD-E	VD-P	VD-E	VD-P	VD-E	VD-P	VD-E
A1	10.76 ± 0.35 ^a^	8.99 ± 0.29 ^b^	0.405 ± 0.002 ^a^	0.291 ± 0.002 ^b^	4.4 ± 0.6 ^a^	4.2 ± 0.9 ^a^	1.1 ± 0.2 ^a^	1.1 ± 0.2 ^a^
A2	11.11 ± 0.89 ^a^	7.69 ± 0.48 ^b^	0.357 ± 0.001 ^a^	0.296 ± 0.002 ^b^	3.2 ± 0.4 ^a^	3.0 ± 0.6 ^a^	0.9 ± 0.2 ^a^	0.9 ± 0.1 ^a^
A3	7.96 ± 0.76 ^a^	9.03 ± 0.34 ^a^	0.294 ± 0.004 ^b^	0.313 ± 0.003 ^a^	4.0 ± 0.6 ^a^	4.1 ± 0.5 ^a^	0.8 ± 0.3 ^a^	0.9 ± 0.2 ^a^
A4	7.51 ± 0.55 ^a^	8.04 ± 0.70 ^a^	0.231 ± 0.004 ^a^	0.240 ± 0.005 ^a^	4.0 ± 0.6 ^a^	3.9 ± 0.4 ^a^	0.9 ± 0.2 ^a^	1.0 ± 0.2 ^a^
A5	6.26 ± 0.89 ^a^	6.62 ± 0.16 ^a^	0.176 ± 0.004 ^a^	0.166 ± 0.001 ^b^	3.5 ± 0.5 ^a^	3.9 ± 0.8 ^a^	0.9 ± 0.2 ^a^	0.9 ± 0.2 ^a^

* MC—moisture content (%); a_w_ water activity; SF—shear force (kg); PF—penetration force (kg); A—apricot; VD-P—vacuum dryer with a vacuum pump; VD-E—vacuum dryer with an ejector system; Means that do not share a letter are significantly different (*p* < 0.05); Letters ^a^ and ^b^—differences between the sample dried at the same conditions but with a different vacuum dryer (vacuum pump (VD-P) and ejector system (VD-E)).

**Table 7:** Experimentally obtained values of total phenolic, flavonoid and monomeric anthocyanin content, antioxidant activity (FRAP, DPPH and ABTS assay) of apricot samples dried using VD-P and VD-E.

*	TPC		TFC		FRAP		DPPH		ABTS	
	VD-P	VD-E	VD-P	VD-E	VD-P	VD-E	VD-P	VD-E	VD-P	VD-E
A1	227.54 ± 2.4 ^a^	198.66 ± 2.8 ^b^	395.59 ± 7 ^a^	387.15 ± 5 ^a^	1.81 ± 0.01 ^a^	1.77 ± 0.01 ^b^	5.42 ± 0.11 ^a^	5.29 ± 0.02 ^a^	4.83 ± 0.08 ^a^	4.67 ± 0.08 ^a^
A2	303.12 ± 5.5 ^a^	286.97 ± 1.5 ^b^	431.27 ± 6 ^a^	386.39 ± 3 ^b^	2.08 ± 0.03 ^a^	1.92 ± 0.02 ^b^	6.84 ± 0.04 ^a^	6.18 ± 0.09 ^b^	7.22 ± 0.12 ^a^	6.80 ± 0.07 ^b^
A3	268.96 ± 0.5 ^b^	290.11 ± 2.3 ^a^	438.96 ± 7 ^a^	428.34 ± 4 ^a^	1.82 ± 0.00 ^b^	1.92 ± 0.01 ^a^	6.09 ± 0.02 ^b^	6.89 ± 0.11 ^a^	6.10 ± 0.09 ^b^	6.98 ± 0.10 ^a^
A4	235.08 ± 1.9 ^b^	255.30 ± 1.9 ^a^	401.13 ± 6 ^a^	386.09 ± 7 ^b^	1.85 ± 0.01 ^a^	1.86 ± 0.02 ^a^	5.49 ± 0.06 ^b^	5.78 ± 0.00 ^a^	5.71 ± 0.05 ^a^	5.33 ± 0.00 ^b^
A5	228.41 ± 3.1 ^b^	303.30 ± 2.1 ^a^	392.26 ± 6 ^a^	377.68 ± 3 ^b^	1.87 ± 0.01 ^b^	1.96 ± 0.01 ^a^	5.52 ± 0.05 ^a^	5.44 ± 0.07 ^a^	5.33 ± 0.04 ^a^	5.17 ± 0.06 ^b^

* TPC—total phenolic content (mg GAE/100 g DW); TFC—total flavonoid content (mg CE/100 g DW); TMAC—total monomeric anthocyanin content (mg CGE/100 g DW); FRAP assay (mg Fe^2+^/g DW); DPPH assay (mg Trolox/g DW); ABTS assay (mg Trolox/g DW); Means that do not share a letter are significantly different (*p* < 0.05); Letters ^a^ and ^b^—differences between the sample dried at the same conditions but with a different vacuum dryer (vacuum pump (VD-P) and ejector system (VD-E)).

## Data Availability

Data is contained within the article.
